# Supplementary material for: Phagotrophic Protists and Their Associates: Evidence for Preferential Grazing in an Abiotically Driven Soil Ecosystem
Source: Microorganisms. 2021 Jul 21;9(8):1555. doi: 10.3390/microorganisms9081555 (PMC8398437; doi:10.3390/microorganisms9081555)
Supplement: Supplementary file 1 [file microorganisms-09-01555-s001.zip › SupplementalFigs1&2.pdf]

Figure S1

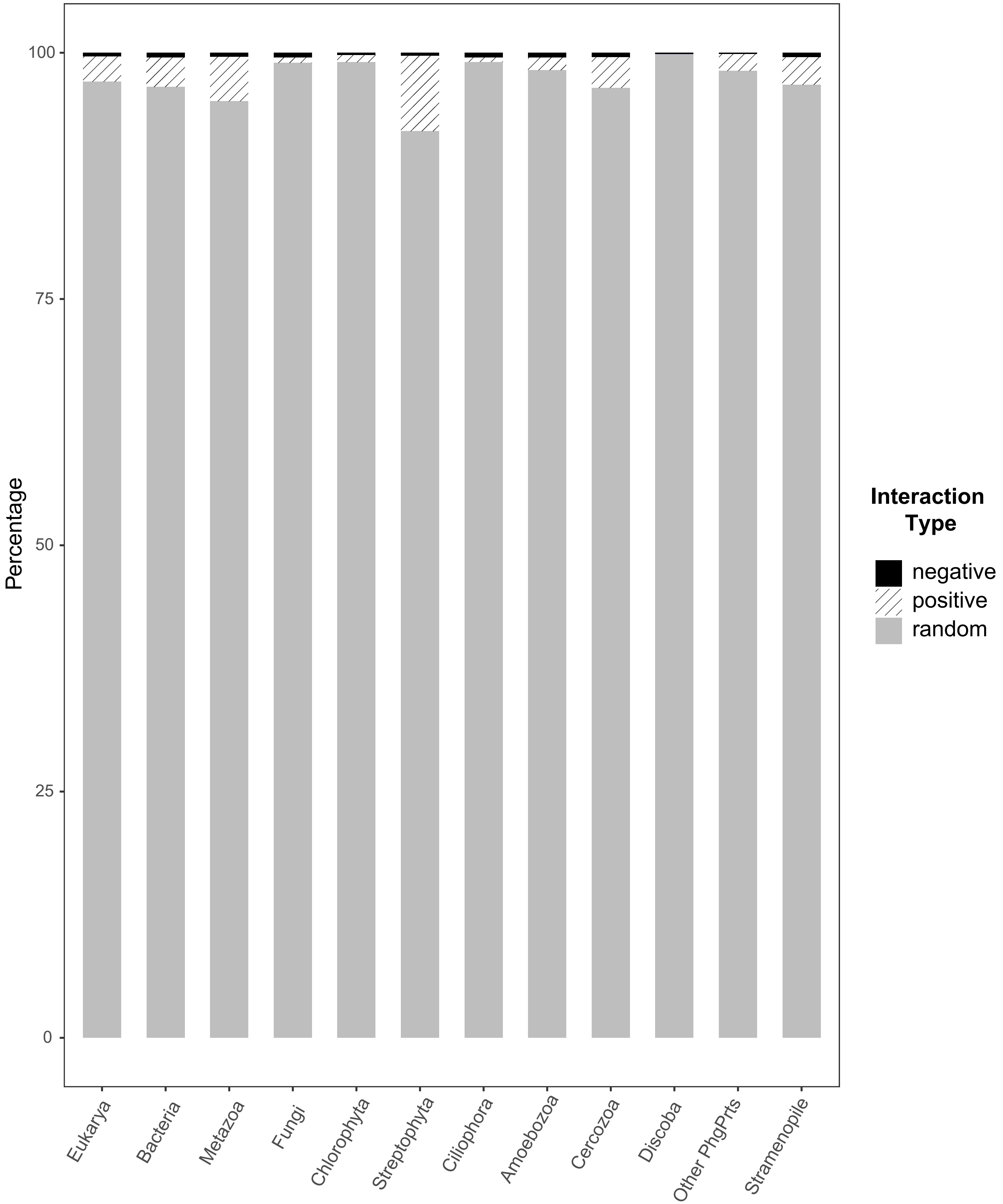

Fig. S1 – Evidence for non-random co-occurrence between MDV taxa. Proportion of associations among OTUs of all biotic groups (x-axis) given by the pairwise analysis of co-occurrence. Gray represents statistically random co-occurrence (significance level of 5%), diagonal lines represent statistically non-random associations of aggregate pairs, and black represent statistically non-random associations of segregate pairs. 'Other phagotrophic protists' are those outside the major groups Cercozoa, Ciliophora, Amoebozoa, and Discoba (e.g., Apusomonadida and Telonemia).

Figure S2

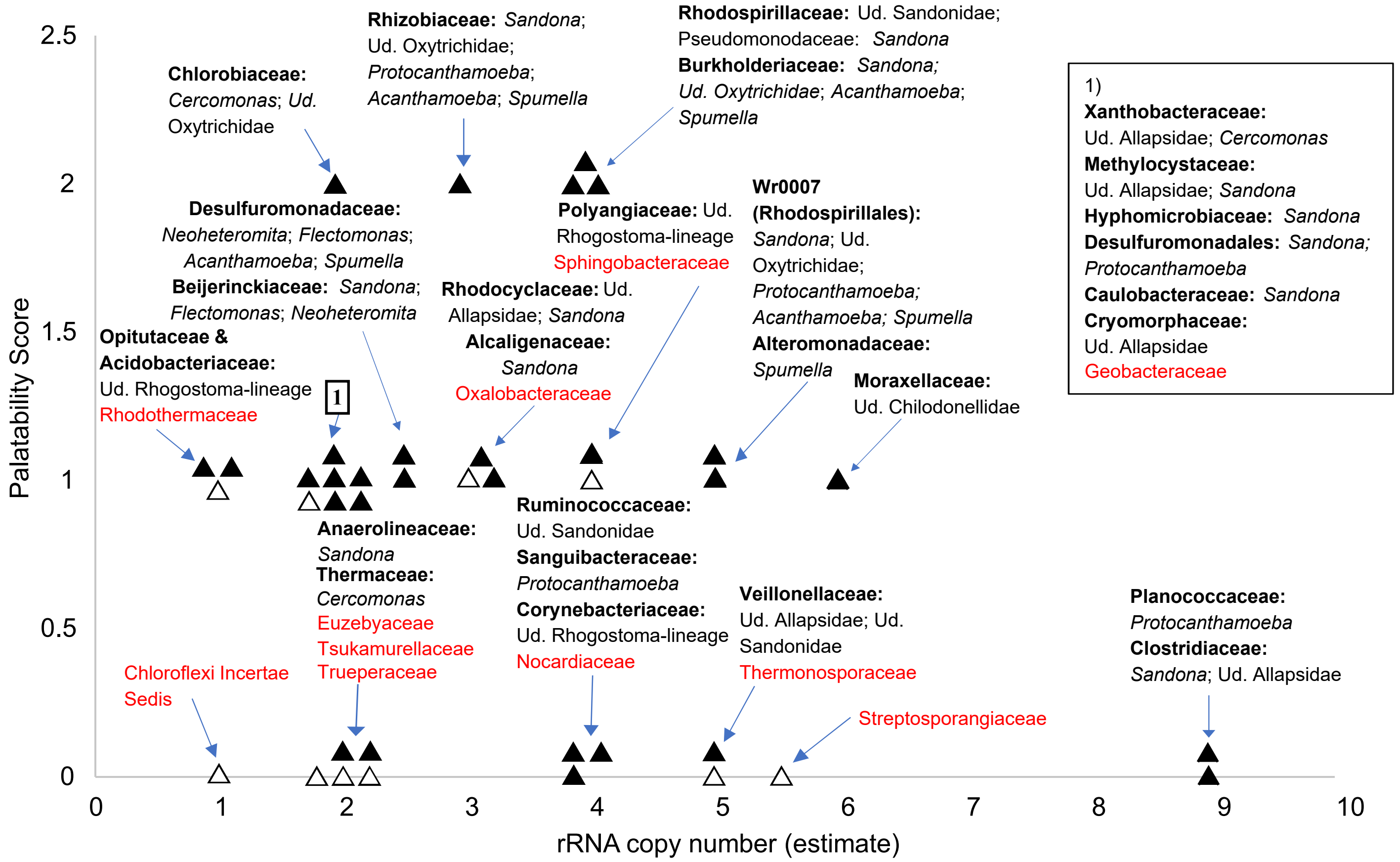

Fig. S2 – A reproduction of the palatability chart in Figure 4, except phagotrophic protist icons have been replaced with protist genera and bacterial families. Names are organized by clusters. Families in red only associate negatively.
